# Supplementary material for: Differences in small noncoding RNAs profile between bull X and Y sperm
Source: PeerJ. 2020 Sep 18;8:e9822. doi: 10.7717/peerj.9822 (PMC7505075; doi:10.7717/peerj.9822)
Supplement: Supplemental Information 1 [file peerj-08-9822-s012.pdf]

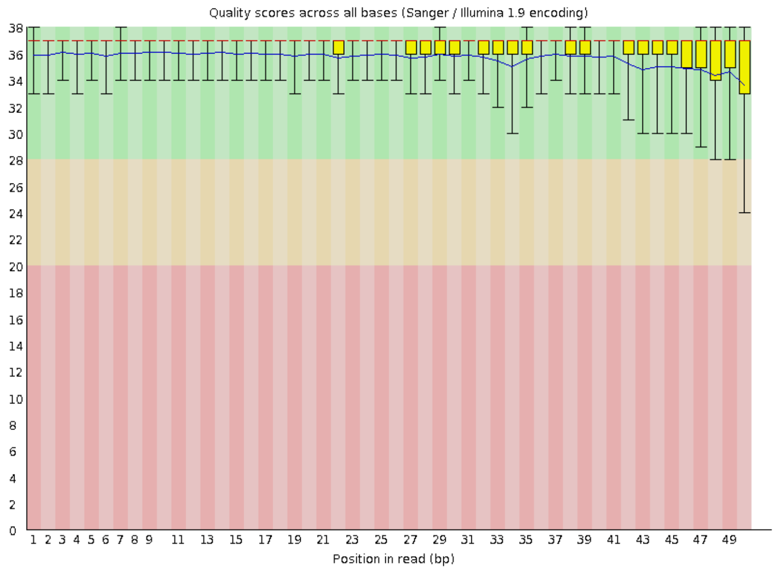


X4069

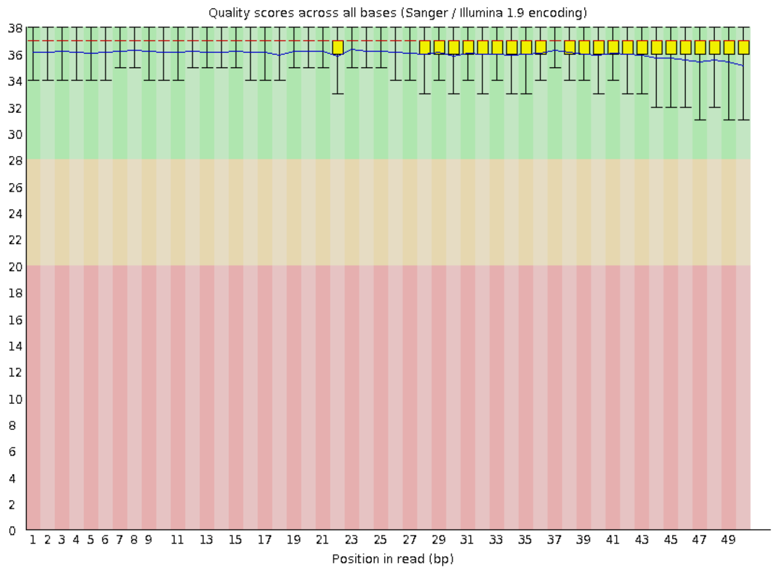


X4118

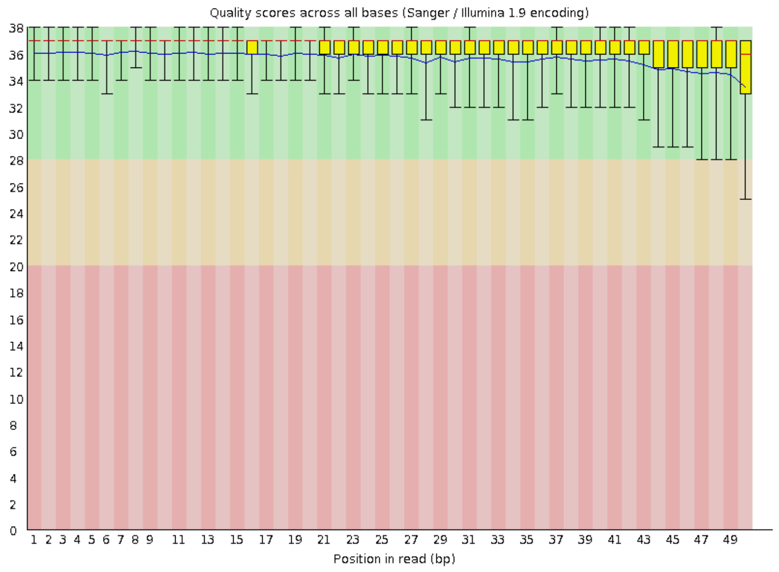


X4131

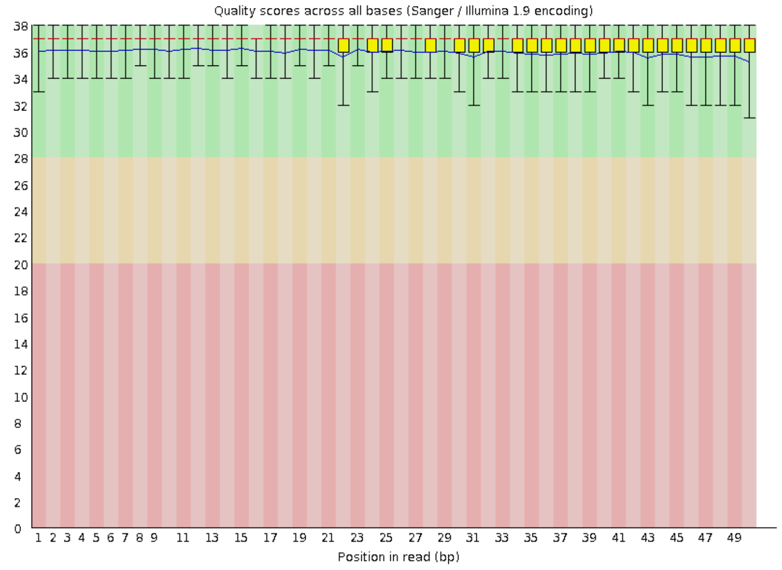


Y4069

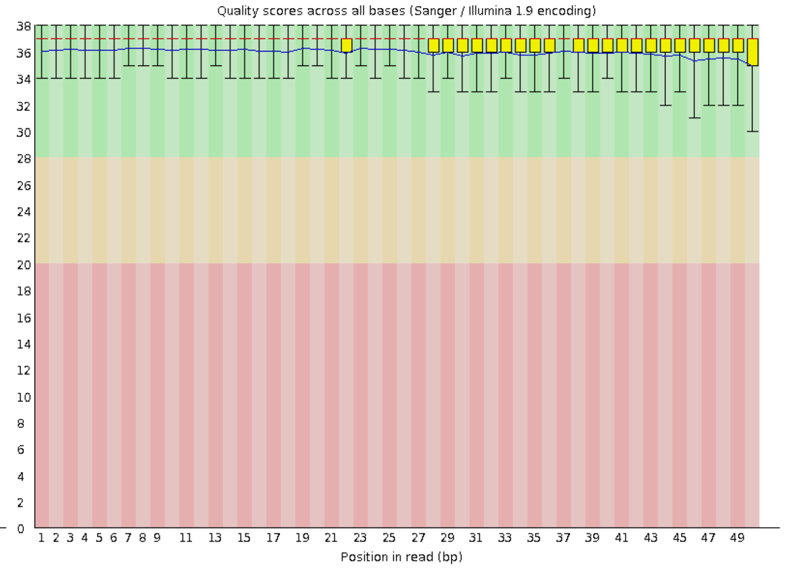


Y4118

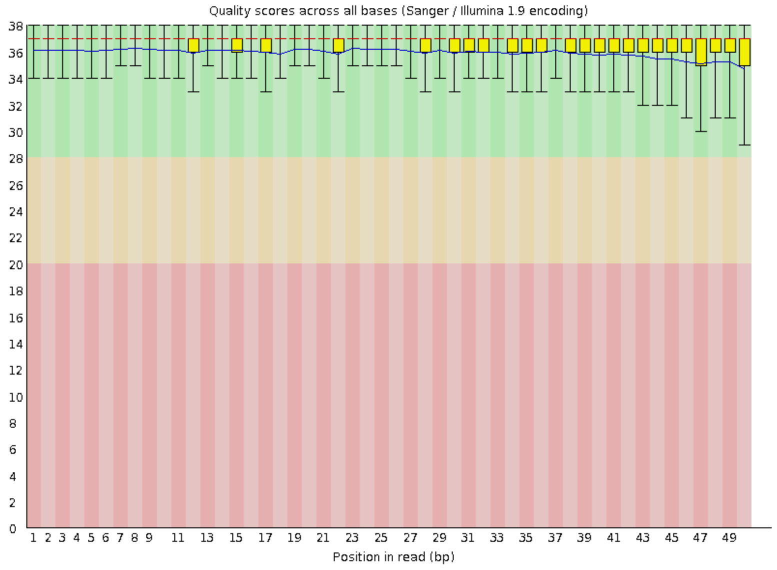


Y4131

The plot of per base sequence quality of raw sequencing data. The y-axis on the graph shows the quality scores. The higher the score the better the base call. The background of the graph divides the y axis into very good quality calls (green), calls of reasonable quality (orange), and calls of poor quality (red).

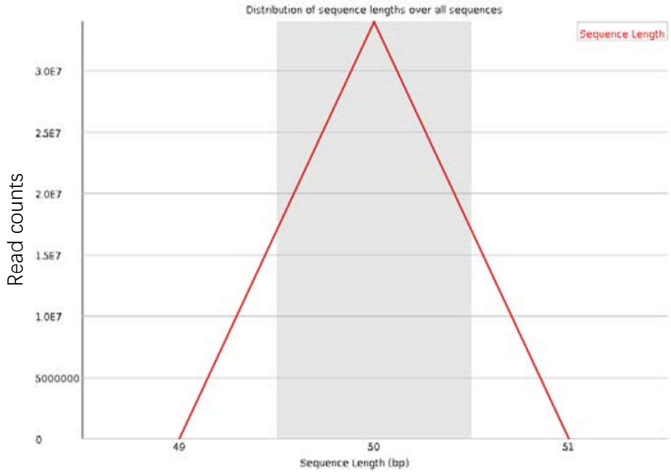


X4069

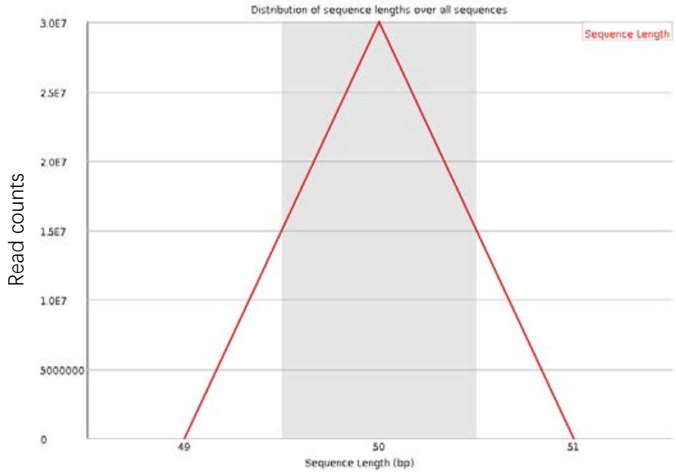


X4118

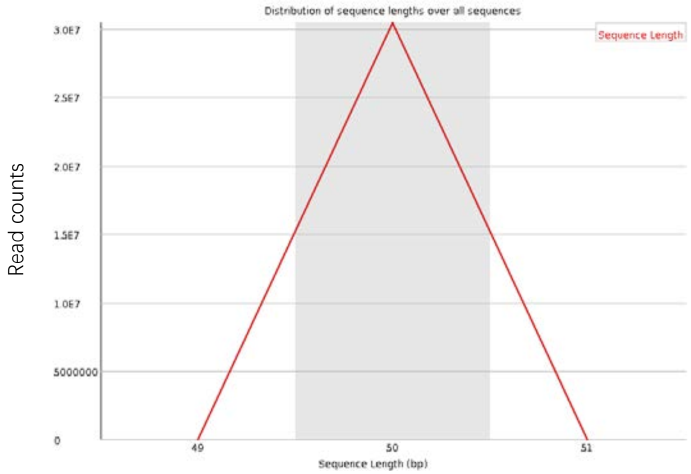


X4131

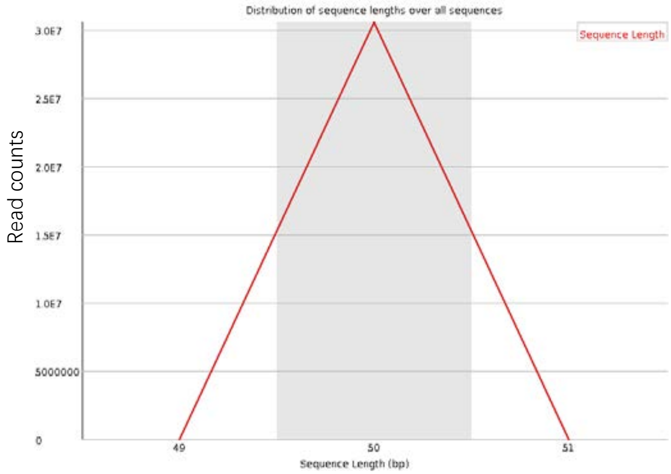


Y4069

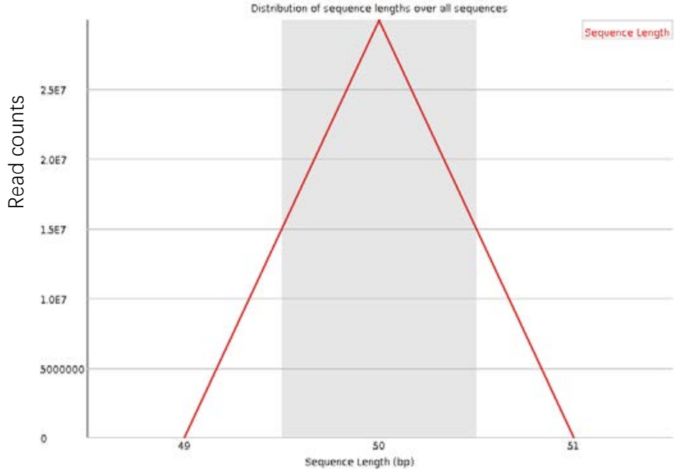


Y4118

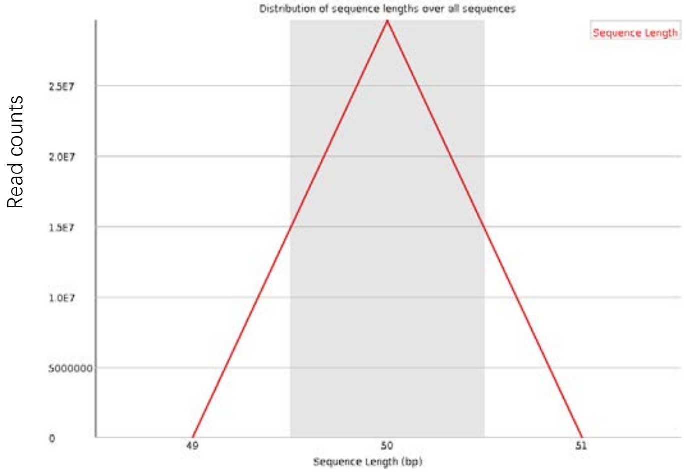


Y4131

The plot of raw sequencing reads distribution.
